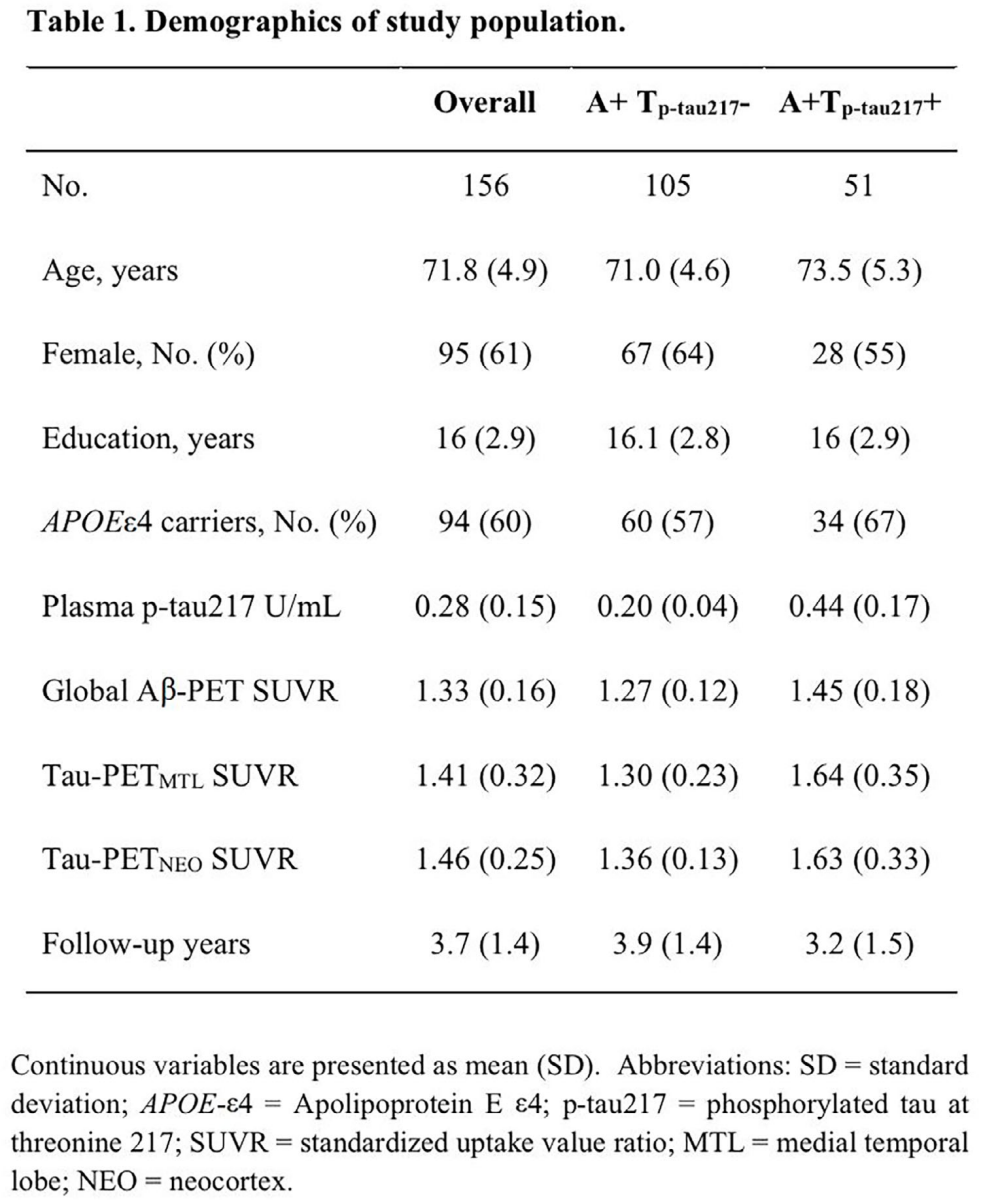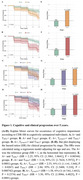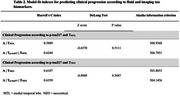# Prognostic utility of *p*‐tau217 and tau PET in Alzheimer's disease

**DOI:** 10.1002/alz70856_107522

**Published:** 2026-01-09

**Authors:** Isabela Just de Jesus Vanni, João Pedro Ferrari‐Souza, Marco Antônio Albini Valer, Lorenzo Fontura Brasil Barcellos, Andrei Bieger, Douglas Teixeira Leffa, Guilherme Povala, Firoza Z Lussier, Wagner S. Brum, Cristiano Aguzzoli, Anderson Corin, Marco Antônio De Bastiani, Giovanna Carello‐Collar, Wyllians Vendramini Borelli, Nesrine Rahmouni, Joseph Therriault, Lydia Trudel, Arthur C. Macedo, Diogo O. Souza, Pamela C.L. Ferreira, Bruna Bellaver, Pedro Rosa‐Neto, Tharick A Pascoal, Eduardo R. Zimmer

**Affiliations:** ^1^ Universidade Federal do Rio Grande do Sul, Porto Alegre, Rio Grande do Sul, Brazil; ^2^ University of Pittsburgh, Pittsburgh, PA, USA; ^3^ Universidade Federal do Rio Grande do Sul, Porto Alegre, RS, Brazil; ^4^ Pontifícia Universidade Católica do Rio Grande do Sul, Porto Alegre, Rio Grande do Sul, Brazil; ^5^ Neurology Department, São Lucas Hospital of PUCRS, Porto Alegre, Rio Grande do Sul, Brazil; ^6^ Universidade Federal de Pelotas, Pelotas, Rio Grande do Sul, Brazil; ^7^ McGill University, Montreal, QC, Canada

## Abstract

**Background:**

Recently proposed biomarker‐based biological staging schemes may improve risk prediction of cognitive impairment in Alzheimer's disease (AD). Evidence demonstrates that tau positron emission tomography (PET) reliably identifies individuals at high risk for clinical progression. While plasma phosphorylated tau at threonine 217 (*p*‐tau217) has been proposed as a cost‐effective biomarker, its added prognostic value to tau PET remains under‐explored. Here, we tested the utility of combining plasma *p*‐tau217 and tau PET for predicting risk of clinical progression in cognitively unimpaired (CU) individuals.

**Method:**

We evaluated 156 CU individuals from the A4 Study placebo group with positron emission tomography (PET) for amyloid‐β (Aβ) plaques ([^18^F]Florbetapir) and tau tangles ([^18^F]Flortaucipir), plasma *p*‐tau217 and longitudinal neuropsychological testing. Aβ‐positive (A+) individuals were classified as *p*‐tau217‐positive (A+T_p‐tau217_+), tau PET‐positive in the medial temporal lobe (A+T_MTL_+) and in the neocortex (A+T_NEO_+). Cutpoints were determined as the mean + 2.0 standard deviations (SD) of the corresponding tau biomarker in Aβ‐negative controls from the LEARN substudy. Time‐to‐event analyses considered clinical progression as a 1 point increase in the Clinical Dementia Rating ‐ Sum of Boxes (CDR‐SB) score, and dichotomized A/T biomarkers were used as predictors.

**Result:**

Demographics of the study population are reported in Table 1. Cox proportional‐hazard models showed a gradual increase in the risk of clinical progression in the A+T_MTL_+ (HR=2.25, *p* = 0.0034) and A+T_NEO_+ (HR=3.14, *p* < 0.0001) groups versus the A+ (reference) group. In analyses incorporating *p*‐tau217 to the models, we found that the A+T_p‐tau217_+ group showed a significantly increased risk for clinical progression compared to A+ group when added to the T_MTL_ model but not to the T_NEO_ model. (Figure 1). Model fit indexes indicated that adding *p*‐tau217 to the models improved the predictive performance of the T_MTL_ model, but not of the T_NEO_ model (Table 2).

**Conclusion:**

We found that *p*‐tau217 positivity does not lead to a clear added prognostic value to tau PET in assessing risk of clinical progression in individuals with preclinical AD, particularly when measuring neocortical tau PET signal. These findings support imaging biomarkers as the primary prognostic tools for predicting cognitive impairment in early AD stages.